# Gene Mutations and Related Molecular Events in Distant Metastasis of Cervical Cancer: A Review

**DOI:** 10.7150/ijms.123727

**Published:** 2026-02-11

**Authors:** Yinghua Guo, Shilong Li, Tingting Liu, Xiaolong Chang, Peiyan Qin, Nan Wang, Yingxiao Jiang, Na Lv, Niannian Li, Furong Hao

**Affiliations:** Weifang People's Hospital, Shandong Second Medical University, Weifang, Shandong 261000, China.

**Keywords:** Cervical cancer, Distant metastasis, Gene mutation, Targeted therapy, HPV integration, microenvironment, epigenetics

## Abstract

Cervical cancer, a serious gynecological malignancy, often leads to poor patient prognosis due to distant metastasis. The metastasis mechanism is not fully understood. This study explores the link between gene mutations and distant metastasis in cervical cancer. *PDGFRA*, *TP53*, and *PIK3CA* mutations significantly influence metastasis. Despite its low incidence, *PDGFRA* mutation is closely tied to lymph node and distant metastasis. *TP53* mutation disrupts p53 protein function, promoting tumor cell proliferation, inhibiting apoptosis, and enhancing metastasis. *PIK3CA* mutation activates the PI3K/Akt pathway, stimulating cell proliferation and migration. Detecting these mutations is crucial for diagnosing distant metastasis. It helps identify high-risk patients early, improving diagnostic accuracy and specificity, and guiding clinical treatment decisions. Targeted therapies for *PDGFRA* and *PIK3CA* mutations can control tumor growth and metastasis but face challenges like drug resistance and high costs. This study offers a new theoretical basis and treatment strategy for cervical cancer, pointing to future research directions. Gene mutation detection enhances early identification of high-risk patients, improving diagnostic accuracy. Targeted therapies for *PDGFRA* and *PIK3CA* mutations control tumor growth but face drug resistance and cost issues. This study provides a new theoretical basis and treatment strategy for cervical cancer, guiding future research.

## Introduction

Cervical cancer (CC) is a major public health concern, ranking as the fourth most common cancer globally in terms of both incidence and mortality among women, with an estimated 660,000 new cases and 350,000 deaths worldwide in 2022, accounting for 22.8 % and 16.0 % of incidence and mortality rates of the global rate, respectively 1. Its incidence rate ranks fourth among female malignant tumors, and its mortality rate also ranks fourth 2. In developing countries, the disease burden of cervical cancer is heavier, with higher morbidity and mortality, which seriously affects women's quality of life and health 3.

Cervical cancer is also a public health issue that cannot be ignored. The incidence of cervical cancer is showing a trend of younger people, which has placed a heavy burden on society and families 4. With the continuous advancement of medical technology, the diagnosis and treatment of early cervical cancer have made significant progress, and the survival rate and quality of life of patients have been improved to a certain extent. However, for patients with cervical cancer with distant metastasis, the prognosis is still extremely poor.

Distant metastasis is a key factor leading to poor prognosis in patients with cervical cancer 5. Once cancer cells metastasize to distant organs, such as the lungs, liver, bones, and brain, not only will the difficulty of treatment increase significantly, but the patient's 5-year survival rate will also drop significantly to only about 10%-15% 6. Patients with cervical cancer that develops distant metastasis often need to receive more complex and intensive treatments, such as chemotherapy, radiotherapy, targeted therapy 7. These treatments not only bring heavy physical burden and psychological pressure to patients, but also lead to a substantial increase in medical expenses, bringing heavy economic burden to families and society.

At present, the mechanism of distant metastasis of cervical cancer has not been fully clarified. In-depth research on the mechanism of distant metastasis of cervical cancer and finding effective predictive markers and therapeutic targets are of great significance for improving the prognosis of cervical cancer patients. Gene mutation plays a key role in the occurrence, development and metastasis of tumors. Studies have shown that multiple gene mutations are closely related to distant metastasis of cervical cancer 8. Therefore, this study aims to explore the relationship between gene mutation and distant metastasis of cervical cancer and provide new theoretical basis and treatment strategies for the prevention and treatment of cervical cancer.

## Metastatic Pathways of Cervical Cancer

Cervical cancer is a malignant tumor that seriously threatens women's health. Its metastasis pathways mainly include direct spread, lymphatic metastasis and hematogenous metastasis 5. Different metastatic pathways play different roles in the development of cervical cancer and have an important impact on the progression and prognosis of patients.

### Direct spread

**Figure 1:**
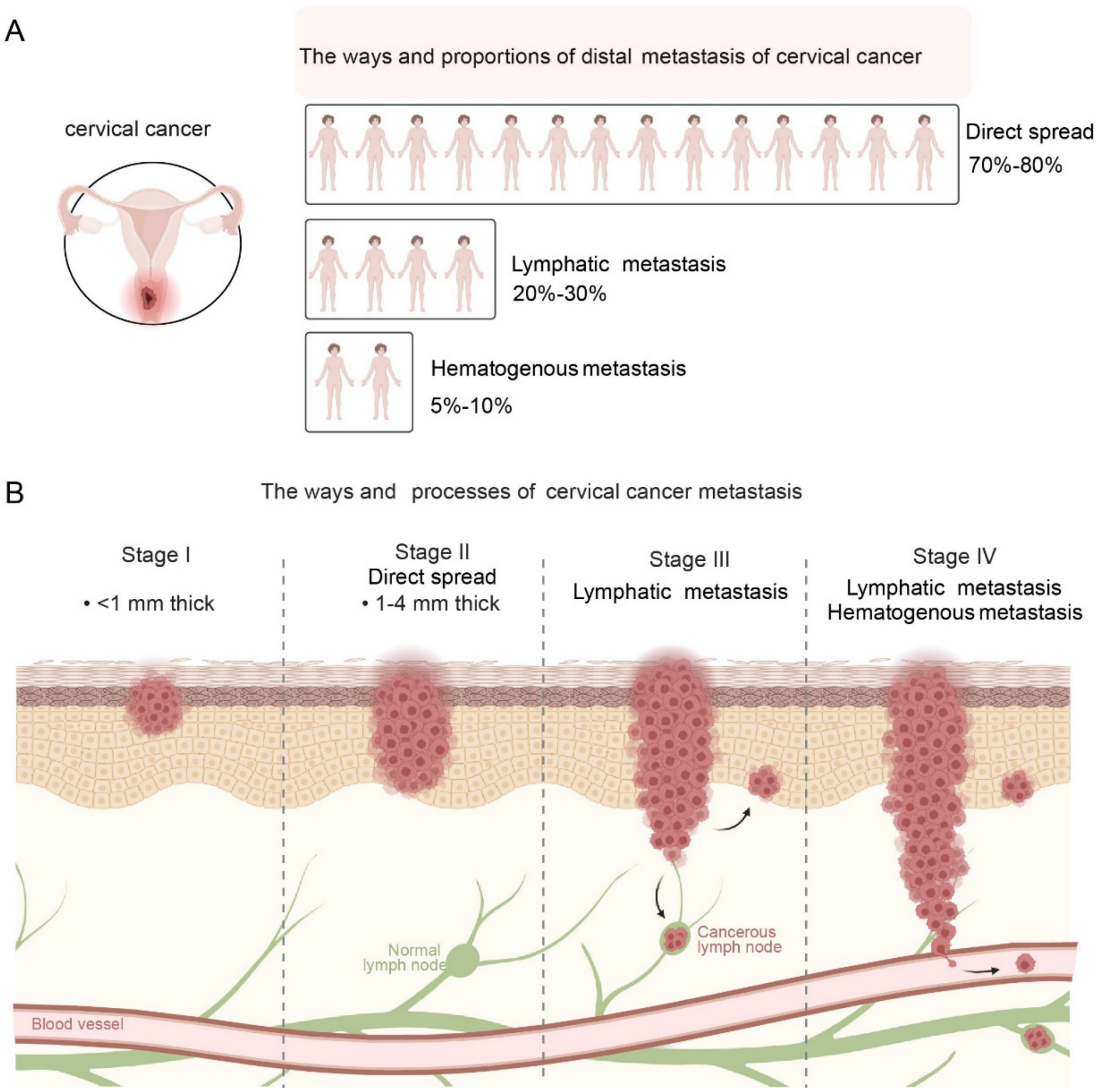
Metastasis pathways of cervical cancer. A: Distribution of metastatic modes: Direct spread is the predominant pathway, followed by lymphatic and hematogenous metastasis. B: Stepwise progression of metastasis: The diagram illustrates the invasion depth and spread from Stage I (local confinement) to Stage IV, where cancer cells disseminate to distant organs via lymphatic and vascular systems.

Direct spread is the most common mode of metastasis of cervical cancer, accounting for about 70%-80% of all metastatic cases 9 (Figure 1A). Cancer cells can directly invade adjacent tissues and organs, such as spreading downward to the vaginal wall, causing irregular vaginal bleeding, increased secretions, etc.; they can also spread upward to the uterine cavity, causing complications such as endometritis and pyometra 10. It invades the main ligaments and paracervical and paravaginal tissues on both sides, and even to the pelvic wall, causing lumbosacral pain, lower limb edema, etc. In the late stage, cancer cells may also invade the bladder forward, causing urinary system symptoms such as frequent urination, urgency, pain, and hematuria; and invade the rectum backward, causing intestinal symptoms such as constipation, bloody stools, and tenesmus. The scope and degree of direct spread are closely related to factors such as the size, location, and degree of differentiation of the tumor. The larger the tumor, the closer it is to the surrounding tissues and organs, and the lower the degree of differentiation, the higher the risk of direct spread.

### Lymphatic metastasis

Lymphatic metastasis is one of the common metastatic pathways of cervical cancer, accounting for about 20%-30% of all metastatic cases 11 (Figure 1A). When the cancer invades the lymphatic vessels, the cancer cells will enter the local lymph nodes along with the lymph fluid 12. Lymphatic metastasis of cervical cancer usually first affects the first-level lymph nodes, including parauterine, obturator, internal iliac, external iliac, common iliac, and presacral lymph nodes 13. These lymph nodes are located in the pelvic cavity and are the first stop for lymphatic metastasis of cervical cancer 14. If the disease progresses further, cancer cells will metastasize to the secondary lymph nodes, such as the deep and superficial inguinal lymph nodes, para-aortic lymph nodes 15. The occurrence of lymph node metastasis is related to factors such as tumor stage, pathological type, and degree of differentiation of tumor cells. The later the tumor stage, the more adenocarcinoma or undifferentiated carcinoma the pathological type, and the less differentiated the tumor cells are, the greater the possibility of lymph node metastasis (Figure 1B).

### Hematogenous metastasis

Hematogenous metastasis is relatively uncommon in cervical cancer, accounting for only 5%-10% of all metastatic cases 16 (Figure 1A). Usually occurs in the late stage of cervical cancer, cancer cells spread through the blood circulation to various organs throughout the body, such as the lungs, liver, bones, brain 17. The occurrence of hematogenous metastasis is closely related to the biological characteristics of tumor cells, the body's immune status, angiogenesis and other factors. Tumor cells have strong invasion and migration capabilities, the body's immune function is low, and there is abundant angiogenesis in tumor tissue, which may increase the risk of hematogenous metastasis 18. When cervical cancer metastasizes through the blood, the patient will experience symptoms of the corresponding metastatic organ. Metastasis to the lungs can cause coughing, hemoptysis, chest pain, dyspnea, etc. Metastasis to the liver can cause liver pain, jaundice, ascites, abnormal liver function, etc. Metastasis to the bones can cause bone pain, pathological fractures, spinal cord compression, etc. Metastasis to the brain can cause headaches, dizziness, vomiting, visual impairment, hemiplegia (Figure 1B).

Among, lymphatic metastasis and hematogenous metastasis are both types of distal metastasis. Distal metastasis refers to the spread of cancer cells to organs or tissues far away from the primary tumor site through blood or lymphatic metastasis 19, such as the lungs, liver, bones, and brain, through hematogenous metastasis or lymphatic metastasis. These organs and tissues are essential for maintaining the normal physiological functions of the human body. Once invaded by cancer cells, they will seriously affect the patient's quality of life and health. Compared with local metastasis, distant metastasis is more difficult to treat and the patient's prognosis is worse 20. Because the cancer cells have spread to many parts of the body, it is difficult to completely remove them through surgery 21. Usually, comprehensive treatment methods such as chemotherapy, radiotherapy, and targeted therapy are required, but the effects of these treatments are often limited, and the 5-year survival rate of patients is low.

## Types of Gene Mutations Associated with Distant Metastasis of Cervical Cancer

### *PDGFRA* gene mutations are associated with the distal metastasis of cervical cancer

The *PDGFRA* gene is located on human chromosome 4q21.3 and contains 23 exons 22. Encodes platelet-derived growth factor receptor α (*PDGFRα*), a member of the tyrosine kinase receptor family 23. *PDGFRα* is mainly expressed in tumor cells and is involved in cell proliferation, migration, and angiogenesis 24. When the *PDGFRA* gene binds to the corresponding ligand platelet-derived growth factor (*PDGF*), it activates the phosphorylation pathways of phosphatidylinositol, cAMP and various proteins, thereby regulating cell division and proliferation 23. Under normal physiological conditions, the expression and function of the *PDGFRA* gene are strictly regulated to maintain normal cell growth and differentiation. During tumorigenesis, abnormal activation of the *PDGFRA* gene can lead to tumorigenesis and promote tumor angiogenesis 25.

There are various types of *PDGFRA* gene mutations, including point mutations, insertion/deletion mutations, and gene amplification 26. Point mutations are the most common type of mutation, accounting for more than 50% of *PDGFRA* gene mutations 27. Point mutations can cause the amino acids encoded by genes to be replaced, thus affecting the structure and function of proteins 28. In gastrointestinal stromal tumors, common *PDGFRA* point mutation sites include D842V mutation in exon 18 29. These mutations lead to enhanced tyrosine kinase activity of *PDGFRα* protein, which enables it to continuously activate downstream signaling pathways in the absence of ligand binding, promoting tumor cell proliferation and survival 30. Insertion/deletion mutations can lead to changes in gene structure, thereby affecting gene expression and function 31. In some tumors, insertion/deletion mutations in the *PDGFRA* gene can change the structure of the *PDGFRα* protein, making it unable to bind to ligands or activate downstream signaling pathways normally, thereby affecting the biological behavior of tumor cells 32. Gene amplification may lead to increased gene expression levels. Studies have found that the amplification of the *PDGFRA* gene is associated with the occurrence and development of cervical cancer 33. Gene amplification may lead to increased expression of *PDGFRA* protein, thereby affecting the growth and proliferation of cervical cancer cells 34.

In cervical cancer, although the incidence of *PDGFRA* gene mutation is relatively low, about 5%-10%, it is associated with the development and prognosis of cervical cancer 35. These mutations are mainly concentrated in the specific exon 18 region 36. Studies have shown that *PDGFRA* gene mutations are associated with the risk of cervical cancer metastasis 35. Cervical cancer patients with *PDGFRA* gene mutations are more likely to develop lymph node metastasis and distant metastasis. Its mechanism of action mainly includes the following aspects: In terms of cell proliferation, *PDGFRA* gene mutations can lead to abnormal activation of receptor signaling pathways 24, Continuously activate the downstream mitogen-activated protein kinase (MAPK) signaling pathway and phosphatidylinositol-3-kinase (PI3K)/AKT signaling pathway, etc., to promote the proliferation and survival of tumor cells 37. In terms of cell migration and invasion, the mutated *PDGFRα* protein affects the interaction between tumor cells and the matrix 24, activates signaling molecules involved in cell motility and invasion, such as Rho GTPases and matrix metalloproteinases (MMPs) 38, This promotes the invasion and metastasis of tumor cells. *PDGFRA* gene mutations may also affect the response of tumor cells to treatment, resulting in reduced sensitivity of tumor cells to certain chemotherapy drugs or targeted therapy drugs, thereby affecting prognosis assessment and treatment selection 39. Studies have shown that cervical cancer patients with *PDGFRA* gene mutations have increased resistance to chemotherapy drugs such as cisplatin and poor treatment effects 40.

### *TP53* - related mutations are associated with the distal metastasis of cervical cancer

The *TP53* gene is located on human chromosome 17p13.1, is approximately 20 kb in length, and contains 11 exons 41. It encodes a 53kDa nuclear phosphoprotein p53, which is an important tumor suppressor gene. 42. Under normal physiological conditions, p53 protein plays a variety of key biological functions in cells and plays a vital role in maintaining normal cell growth, differentiation and genome stability.

In cervical cancer, *TP53* gene mutation is common, and about 30%-50% of cervical cancer patients have *TP53* gene mutation 43. There are various types of *TP53* gene mutations, including point mutations, deletions, insertions 44. Point mutations are the most common type of mutation, accounting for more than 80% of *TP53* gene mutations. Point mutations usually occur in the highly conserved exon regions 5-8 of the *TP53* gene 45. These regions encode amino acids that are essential for the structure and function of the p53 protein. Different types of *TP53* gene mutations can lead to changes in the structure and function of p53 protein, thereby affecting its regulatory effects on cell cycle regulation, DNA repair, and cell apoptosis, thereby promoting the occurrence and development of cervical cancer 46.

*TP53* gene mutations can lead to loss of function or abnormality of p53 protein, making it unable to properly regulate the cell cycle 47. Mutant p53 protein cannot effectively activate the expression of the p21 gene, resulting in dysfunctional cell cycle checkpoints 48. The cells cannot stop proliferating in time to repair damaged DNA, allowing the cells to continue to enter the cell cycle for division, increasing the risk of gene mutation and chromosomal instability, thereby promoting the proliferation of tumor cells. Studies have shown that in cervical cancer cells carrying *TP53* gene mutations, the expression level of the p21 gene is significantly reduced, the cell cycle process is accelerated, and the cell proliferation ability is significantly enhanced 49.

*TP53* gene mutations also affect the cell's DNA repair ability. Mutated p53 proteins cannot normally participate in the DNA repair process, resulting in a decrease in the cell's ability to repair DNA damage 50. This makes it impossible for damaged DNA to be repaired in time, further accumulating gene mutations and promoting the occurrence and development of tumors. Studies have found that in cervical cancer tissues with *TP53* gene mutations, the expression and activity of DNA damage repair-related proteins are significantly reduced, the efficiency of DNA damage repair is reduced, and genomic instability is increased 51.

In terms of cell apoptosis, *TP53* gene mutations can cause p53 protein to lose its ability to induce cell apoptosis, leading to cell escape from apoptosis, thus allowing tumor cells to continue to survive and proliferate 52. The mutant p53 protein cannot effectively regulate the expression of pro-apoptotic and anti-apoptotic genes, which makes the apoptotic signaling pathway in the cell unable to be activated normally and reduces the sensitivity of the cell to apoptosis 53. Studies have shown that in cervical cancer cells with *TP53* gene mutations, the expression levels of pro-apoptotic genes such as Bax are reduced, the expression levels of anti-apoptotic genes such as Bcl-2 are increased, cell apoptosis is inhibited, and the survival and proliferation abilities of tumor cells are enhanced 54.

*TP53* gene mutation is also closely related to distant metastasis of cervical cancer. Mutant p53 protein not only loses its inhibitory effect on tumor cells, but also may acquire some new cancer-promoting functions that promote the invasion and metastasis of tumor cells 55. Studies show that cervical cancer patients with *TP53* gene mutations are more likely to have distant metastasis and have a poor prognosis 56. Its mechanism of action may include: mutant p53 protein can enhance the invasion and metastasis ability of tumor cells by regulating some genes and signaling pathways related to tumor cell invasion and metastasis, such as matrix metalloproteinases (MMPs) and epithelial-mesenchymal transition (EMT) related genes. MMPs are a class of proteases that can degrade the extracellular matrix and play an important role in the invasion and metastasis of tumor cells 57. Mutant p53 protein can upregulate the expression of MMPs, promote the degradation of extracellular matrix, and provide conditions for the migration and invasion of tumor cells 58. EMT refers to the process in which epithelial cells lose polarity and intercellular connections and acquire mesenchymal cell characteristics, which can enable tumor cells to acquire stronger migration and invasion capabilities 59. Mutant p53 protein can induce EMT by regulating the expression of EMT-related genes E-cadherin, N-cadherin, and Vimentin, thereby promoting the invasion and metastasis of tumor cells 60.

Mutant p53 regulates EMT primarily through three pathways: direct regulation, signaling pathway interaction, and epigenetic modification. Firstly, it can directly bind to the promoters of EMT-promoting factors such as SNAI1 and TWIST1, enhancing their activity, or weaken the effects of EMT-repressing factors such as ZEB1 by competitive binding sites, thereby regulating the expression of epithelial and stromal markers 61. Secondly, it can interact with signaling pathways such as PI3K/Akt, TGF-β/Smad, and Wnt/β-catenin, for example, binding to the PI3K catalytic subunit to enhance its activity and stabilize Snail, or enhancing TGF-β-mediated Smad phosphorylation, indirectly promoting the EMT process 62. Thirdly, it can also recruit histone-modifying enzymes such as HDACs and EZH2, or upregulate DNMTs activity, epigenetically modifying EMT-related genes such as E-cadherin, inhibiting epithelial marker gene transcription or silencing EMT-repressing genes, ultimately achieving EMT regulation 63.

### *PIK3CA* - related mutations are associated with the distal metastasis of cervical cancer

The *PIK3CA* gene is a key proto-oncogene located on chromosome 3 and has 20 exons 64. This gene belongs to the PI3K-Akt signaling pathway and is primarily responsible for encoding the p110α protein, a catalytic subunit of the PI3K enzyme 65. During normal cell signaling, the PI3K enzyme is activated by cell surface receptors 66. By phosphorylating other proteins, it triggers a series of intracellular signal transduction, thereby regulating various biological processes such as cell growth, proliferation, survival, and metabolism 67.

*PIK3CA* gene mutations can lead to abnormalities in the structure and function of the p110α protein, causing the PI3K enzyme to be in a state of continuous activation, thereby enhancing the conduction of intracellular signals and causing disorders in the PI3K/Akt signaling pathway 68. Abnormal activation of this pathway will cause abnormal cell proliferation, resistance to apoptosis, promotion of angiogenesis, and enhancement of cell migration and invasion, ultimately leading to the occurrence and development of tumors 69. About 80% of *PIK3CA* mutations occur in two hotspots: the helical region and the kinase region 70. The three most common mutations are H1047R in exon 20, E542K and E545K in exon 9 71.

In cervical cancer, *PIK3CA* gene mutation also has a certain incidence rate, about 10%-20% 72. Studies have shown that *PIK3CA* gene mutations are closely related to distant metastasis of cervical cancer. A study of 100 patients with cervical cancer found that the frequency of *PIK3CA* gene mutations in patients with distant metastasis was significantly higher than that in patients without metastasis, at 30% and 10% respectively 73. This suggests that *PIK3CA* gene mutation may be an important risk factor for distant metastasis of cervical cancer 74.

*PIK3CA* gene mutation activates the PI3K/Akt signaling pathway, which significantly affects the proliferation, survival, and migration of cervical cancer cells 75. In terms of cell proliferation, activated Akt protein can phosphorylate a variety of downstream substrates 76, such as mammalian target of rapamycin (mTOR), which promotes protein synthesis and cell cycle progression, thereby accelerating the proliferation of cervical cancer cells. The study found that in cervical cancer cell lines carrying *PIK3CA* gene mutations, cell proliferation was significantly accelerated, and the expression of proliferation-related proteins such as cyclin D1 was significantly upregulated 77. In terms of cell survival, Akt protein can inhibit the activity of apoptosis-related proteins Bad and Caspase, thereby enhancing the survival ability of cervical cancer cells 78. The experiment showed that when the PI3K/Akt signaling pathway was inhibited, the apoptosis rate of cervical cancer cells carrying the *PIK3CA* gene mutation increased significantly, indicating that the activation of this signaling pathway plays an important protective role in cell survival 79. In terms of cell migration and invasion, activation of the PI3K/Akt signaling pathway can regulate cytoskeletal reorganization 80, Promote the expression and secretion of proteins such as matrix metalloproteinases (MMPs), thereby enhancing the migration and invasion ability of cervical cancer cells. Studies have shown that in cervical cancer cells with *PIK3CA* gene mutations, the expression levels of proteins such as MMP-2 and MMP-9 are significantly increased, and the migration and invasion abilities of cells are significantly enhanced 81.

### *BRAF* and *EGFR* gene mutations

In addition to the genes mentioned above, mutations in the *BRAF* and *EGFR* genes also play a significant role in distant metastasis in cervical cancer. The *BRAF* gene, a key molecule in the MAPK signaling pathway, has a hotspot mutation, V600E, which occurs in approximately 3%-5% of cervical cancers 82. This mutation leads to sustained activation of the *BRAF* protein, which in turn activates the downstream MEK/ERK signaling pathway through a phosphorylation cascade, leading to upregulation of epithelial-mesenchymal transition (EMT)-related transcription factors such as Snail and Twist, ultimately enhancing tumor cell migration and invasion 83. Clinical studies have shown that cervical cancer patients with the *BRAF* V600E mutation are more likely to develop lung metastases and have a relatively poor prognosis 84.

*EGFR* (epidermal growth factor receptor) gene mutations are also closely associated with the metastatic potential of cervical cancer, with mutations such as L858R being particularly common 85. Mutated *EGFR* can activate the downstream dual signaling pathways of PI3K/Akt and MAPK through autophosphorylation 86. Activation of the PI3K/Akt pathway enhances cell survival and promotes the secretion of matrix metalloproteinases (such as MMP-2 and MMP-9) 87. Sustained activation of the MAPK pathway further accelerates cell cycle progression and EMT, collectively enhancing tumor cell migration. In vitro studies have demonstrated that targeted inhibitors targeting *EGFR* mutations (such as gefitinib) can specifically block these signaling pathways and significantly inhibit the invasive ability of cervical cancer cells carrying these mutations, providing a potential target for the treatment of these patients 88.

### HPV integration-related gene mutations

Human papillomavirus (HPV) integration into the host genome is a key driver of cervical cancer development. HPV16 integration is particularly common, withHPV integration-related genetic abnormalities have been detected in approximately more than 50% of patients with cervical squamous cell carcinoma 89. The E7 oncoprotein in the HPV genome is a core molecule mediating malignant transformation 90. It directly binds to the retinoblastoma protein (RB1), leading to RB1 functional inactivation 91. This process not only relieves RB1's inhibitory effect on the cell cycle, leading to abnormal cell proliferation, but also indirectly induces the degradation of the tumor suppressor gene *TP53*, impairing the cell's DNA damage repair capacity and apoptosis mechanism.

Notably, genetic abnormalities triggered by HPV integration often synergize with mutations in other driver genes. For example, when HPV16 E7-mediated RB1 inactivation coexists with *PIK3CA* mutations, it can significantly accelerate tumor cell invasion and metastasis through the dual mechanisms of “cell cycle deregulation - accelerated proliferation” and “PI3K/Akt pathway activation - enhanced migration 92.” This synergistic effect of multiple gene abnormalities is not only a key factor in the increased malignancy of cervical cancer but also provides a molecular rationale for the development of combined targeted therapy strategies in clinical practice.

### Other related gene mutations

In addition to the above-mentioned gene mutations, some gene mutations such as *KRAS* and *PTEN* are also associated with distant metastasis of cervical cancer, and they play a potential role in the metastasis of cervical cancer.

The *KRAS* gene is an important member of the RAS gene family, encoding a small GTPase that plays a key molecular switch role in cell signaling pathways 93. Normally, *KRAS* protein is inactive when bound to GDP 94. When stimulated by upstream signals, *KRAS* protein binds to GTP and becomes activated. The activated *KRAS* protein can activate downstream mitogen-activated protein kinase (MAPK) signaling pathways, phosphatidylinositol-3 kinase (PI3K)/AKT signaling pathways 95, regulates biological processes such as cell proliferation, differentiation, survival and migration 96. *KRAS* gene mutations are common in many tumors, which can reduce the GTPase activity of *KRAS* protein, causing it to remain in an activated state and continuously activate downstream signaling pathways, thereby promoting the occurrence and development of tumors 97. In cervical cancer, although the incidence of *KRAS* gene mutation is relatively low, about 5%-10%, studies have shown that this gene mutation is associated with distant metastasis of cervical cancer 98. A study of 150 patients with cervical cancer found that the frequency of *KRAS* gene mutations was significantly higher in patients with distant metastasis than in those without metastasis, at 15% and 5%, respectively 99. Further mechanistic studies have shown that *KRAS* gene mutations promote the proliferation, migration and invasion of cervical cancer cells by activating the MAPK and PI3K/AKT signaling pathways, thereby increasing the risk of distant metastasis of cervical cancer 100. In cervical cancer cell lines carrying *KRAS* gene mutations, cell proliferation was significantly accelerated, and migration and invasion abilities were significantly enhanced. At the same time, the phosphorylation levels of key proteins in downstream signaling pathways, such as ERK and AKT, were significantly increased 101.

Clinically, the detection of *KRAS* mutations has practical guiding value for the diagnosis and treatment of cervical cancer: for patients with locally advanced cervical cancer (LACC), if a *KRAS* mutation is detected preoperatively, the risk of distant metastasis should be considered, and the frequency of postoperative imaging follow-up should be increased 102; while in the treatment of metastatic cervical cancer, *KRAS* mutations may indicate that the patient's sensitivity to platinum-based chemotherapy (such as cisplatin) is reduced—because the continuously activated PI3K/AKT pathway enhances the ability to repair DNA damage, leading to chemotherapy resistance^103^. Such patients may need to prioritize combination therapy (such as PI3K inhibitors) to improve treatment response.

The *PTEN* gene is an important tumor suppressor gene. The *PTEN* protein it encodes has phosphatase activity and can negatively regulate the PI3K/AKT signaling pathway. In normal cells, *PTEN* protein inhibits the activation of the PI3K/AKT signaling pathway by dephosphorylating phosphatidylinositol-3,4,5-triphosphate (PIP3) to phosphatidylinositol-4,5-bisphosphate (PIP2), thereby inhibiting cell proliferation, promoting cell apoptosis, and inhibiting cell migration and invasion 104. When the *PTEN* gene is mutated or missing, the expression or activity of *PTEN* protein decreases, and it cannot effectively inhibit the PI3K/AKT signaling pathway, resulting in the continued activation of the signaling pathway, which in turn promotes the occurrence and development of tumors 105. In cervical cancer, the mutation or deletion of *PTEN* gene also has a certain incidence rate, about 10%-20%. Studies have found that abnormalities in *PTEN* gene are closely related to distant metastasis of cervical cancer 8. A study analyzed 120 patients with cervical cancer and found that the frequency of *PTEN* gene mutation or deletion was significantly higher in patients with distant metastasis than in patients without metastasis, at 25% and 10%, respectively 106. *PTEN* gene abnormalities lead to overactivation of the PI3K/AKT signaling pathway, which may promote the degradation of the extracellular matrix and enhance the migration and invasion ability of cervical cancer cells by upregulating the expression of matrix metalloproteinases (MMPs), thereby promoting the distant metastasis of cervical cancer. In cervical cancer cells with *PTEN* gene deletion, the expression levels of proteins such as MMP-2 and MMP-9 are significantly increased, and the migration and invasion ability of cells are significantly enhanced. At the same time, the phosphorylation level of AKT, a key protein in the PI3K/AKT signaling pathway, is also significantly increased 107.

## Application of Gene Mutation Detection in the Diagnosis of Distant Metastasis of Cervical Cancer

Detection of gene mutations associated with distant metastasis of cervical cancer is of great significance for early detection of potential metastasis risk, improving diagnostic accuracy and specificity, and providing important basis for clinical treatment decision-making.

### Early detection of potential metastasis risks

Gene mutations often appear before distant metastasis of cervical cancer 108. By detecting these gene mutations, patients with a high risk of distant metastasis can be identified in advance. Studies have shown that cervical cancer patients with *PIK3CA* gene mutations have a significantly increased risk of distant metastasis 73. A prospective study of 200 patients with cervical cancer found that during follow-up, 30% of patients with positive *PIK3CA* gene mutations developed distant metastasis within 2 years, while only 10% of patients with negative *PIK3CA* gene mutations developed distant metastasis. This suggests that by detecting *PIK3CA* gene mutations, patients with a high risk of distant metastasis can be identified early, providing an important time window for clinical intervention 109. Early detection of potential metastasis risks can enable patients to receive more aggressive treatments in a timely manner, such as intensive chemotherapy, radiotherapy or targeted therapy, which may delay or prevent the occurrence of distant metastasis and improve the patient's survival rate 110.

### Improving the accuracy and specificity of diagnosis of distant metastasis

Detection of gene mutations can improve the accuracy and specificity of diagnosis of distant metastasis of cervical cancer 111. Traditional imaging examinations and tumor marker detection diagnostic methods have certain limitations 112. Imaging examinations may be difficult to detect tiny metastases and may easily lead to missed diagnosis 113; Tumor marker testing has poor specificity and may be elevated in some benign diseases, leading to false positive results 114. Gene mutation detection has high specificity and can accurately reflect the molecular characteristics of tumor cells, providing a more reliable basis for diagnosis. For some early metastatic lesions that are difficult to diagnose through imaging examinations, combining gene mutation detection results can improve the accuracy of diagnosis. 115. The study showed that when diagnosing patients with suspected cervical cancer lung metastasis, the accuracy of diagnosis increased from 70% to 85% by testing *TP53* gene mutations and lung imaging findings simultaneously 116. This suggests that combining gene mutation detection with traditional diagnostic methods can make up for the shortcomings of traditional methods and improve the accuracy and specificity of diagnosis.

### Provision of bases for clinical treatment decisions

The results of gene mutation detection provide an important basis for clinical treatment decisions. Different types of gene mutations have different effects on the biological behavior and treatment response of cervical cancer. Understanding the patient's gene mutation status can help doctors develop personalized treatment plans and improve the targetedness and effectiveness of treatment. For breast cancer patients with HER2 gene amplification, HER2-targeted therapeutic drugs are used clinically 117. These drugs can specifically bind to HER2 protein and block its signal transduction, thereby inhibiting the growth and metastasis of tumor cells and significantly improving the survival rate and quality of life of patients 118. In cervical cancer, for patients with *PIK3CA* gene mutations, since *PIK3CA* gene mutations activate the PI3K/Akt signaling pathway, making tumor cells more sensitive to PI3K/Akt signaling pathway inhibitors, targeted therapy with PI3K/Akt signaling pathway inhibitors can be considered 119. Studies have shown that in cervical cancer patients with *PIK3CA* gene mutations, the objective response rate of patients treated with PI3K/Akt signaling pathway inhibitors was significantly higher than that of patients who did not use the inhibitors. This shows that choosing the appropriate treatment method based on the results of gene mutation detection can improve the treatment effect and the prognosis of patients 120.

### Gene therapy for cervical cancer

The occurrence of cervical cancer is related to multiple gene abnormalities, including activation of oncogenes and inactivation of tumor suppressor genes 121. Gene therapy aims to correct these gene abnormalities through various means, restore the normal function of cells, and thus inhibit the growth and spread of tumor cells 122.

Gene therapy: Gene replacement: Introducing normal tumor suppressor genes into tumor cells to supplement or replace missing or inactivated tumor suppressor genes and restore their function of inhibiting tumor growth. The p53 gene is an important tumor suppressor gene. In many cervical cancer patients, the p53 gene is mutated or missing 123. By introducing the normal p53 gene into tumor cells, it can induce tumor cell apoptosis and inhibit tumor growth. Gene silencing: Technologies such as RNA interference (RNAi) are used to specifically inhibit the expression of oncogenes 124. For the oncogenes overexpressed in cervical cancer, the E6 and E7 genes of human papillomavirus (HPV), corresponding RNAi molecules were designed. These molecules can effectively reduce the expression levels of these genes, thereby inhibiting the proliferation and survival of tumor cells. Immunogeneous gene therapy: By introducing immune regulatory genes, the body's immune response to tumor cells is enhanced 125. For example, genes encoding cytokines (such as interleukin-2, interferon, etc.) are introduced into tumor cells or immune cells. This can promote the activation and proliferation of immune cells and enhance their killing effect on tumor cells 126.

## Therapeutic Strategies for Distant Metastasis of Cervical Cancer Based on Gene Mutations

Targeted therapy is a treatment method that intervenes in specific molecular targets of tumor cells. It has the advantages of precision, high efficiency and few side effects 127. In the treatment of distant metastasis of cervical cancer, targeted therapy mainly targets gene mutations of *PDGFRA* and *PIK3CA*, which are closely related to distant metastasis of cervical cancer 128. By inhibiting the proteins encoded by these mutant genes or their related signaling pathways, the growth, proliferation, invasion and metastasis of tumor cells can be inhibited.

### Targeted Therapy for *PDGFRA* Gene Mutations

Targeted therapy for *PDGFRA* gene mutations focuses on blocking the *PDGFRA*-mediated signaling pathway through tyrosine kinase inhibitors (TKIs) to inhibit tumor cell proliferation and metastasis. Imatinib, as a first-generation TKI, can competitively bind to the ATP binding site of *PDGFRA*, inhibiting its tyrosine kinase activity, thereby blocking the activation of downstream MAPK and PI3K/AKT pathways 129. In clinical studies of advanced or metastatic cervical cancer, the objective response rate (ORR) of imatinib monotherapy in patients with *PDGFRA* mutations reached 22%-28%, with some patients experiencing tumor volume reduction of more than 30% and significant relief of bone metastasis-related pain symptoms 130. Furthermore, the multi-target TKI sunitinib, by simultaneously inhibiting *PDGFRA* and vascular endothelial growth factor receptor (V*EGFR*), exhibits a synergistic effect in patients with *PDGFRA* mutations and highly vascularized metastases (such as liver metastases)—not only inhibiting tumor cell activity through *PDGFRA*, but also blocking tumor angiogenesis and reducing nutrient supply, resulting in a disease control rate (DCR) that is 15%-20% higher than that of imatinib 131.

However, the clinical benefits of *PDGFRA*-targeted therapy are often limited by the emergence of drug resistance, and different resistance mechanisms are closely related to treatment regimens. Secondary mutations are the main cause of imatinib resistance, with the *PDGFRA* exon 18 D842V mutation being the most typical—this mutation reduces the affinity of imatinib for the receptor by altering the ATP-binding pocket structure of *PDGFRα*, resulting in the drug's inability to effectively inhibit kinase activity 29.Clinical data show that among *PDGFRA*-mutant cervical cancer patients treated with imatinib, approximately 35%-40% were found to have the D842V mutation 6-12 months after treatment 132. The progression-free survival (PFS) of these patients was more than 50% shorter than that of those without the mutation, and the response rate to subsequent treatment was significantly lower 37. In addition, *PDGFRA* gene amplification may also lead to drug resistance. Some patients have an increased *PDGFRA* copy number after long-term treatment, which leads to overexpression of *PDGFRA* protein. Even in the presence of drugs, the pathway can still be activated. The incidence of this type of drug resistance in the sunitinib treatment population is approximately 15%-20% 26. In response to the aforementioned drug resistance, next-generation TKIs such as avatinib have higher inhibitory activity against D842V mutations. In vitro experiments show that its inhibitory efficiency against D842V mutant *PDGFRA* is more than 20 times that of imatinib. Its efficacy has been confirmed in gastrointestinal stromal tumors and it is expected to become a salvage treatment option for cervical cancer patients with *PDGFRA* D842V mutations 29.

### Targeted therapy for *PIK3CA* gene mutations

Targeted therapy for *PIK3CA* gene mutations is based on PI3K inhibitors. By inhibiting the activity of PI3Kα catalytic subunits, it blocks the abnormal activation of the PI3K/AKT/mTOR pathway 133. Alpelisib, as a selective inhibitor of PI3Kα, can specifically bind to mutant PI3Kα and has a weak inhibitory effect on wild-type PI3K 134. Therefore, the incidence of toxic side effects (such as hyperglycemia and rash) is reduced by 20%-25% compared with pan-PI3K inhibitors 135. In clinical trials of *PIK3CA*-mutated metastatic cervical cancer, apelelis monotherapy achieved an ORR of 18%-22% and a median PFS of 5.6-6.2 months. Furthermore, in patients with lung metastases complicated by PI3K/AKT pathway overactivation, its lesion shrinkage rate was significantly higher than that of chemotherapy 136. The pan-PI3K inhibitor BKM120, which inhibits four PI3K subtypes (α, β, γ, and δ), showed advantages in patients with *PIK3CA* mutations and other PI3K subtype abnormalities (such as PI3Kβ activation). *In vitro* experiments showed that it inhibited cervical cancer cell proliferation by 60%-65% and induced 30%-35% apoptosis 137.

The resistance mechanisms to PI3K inhibitors are more complex, with bypass signaling activation and pathway remodeling being the main types. *KRAS* activation is a common bypass pathway in apelelis resistance 138. When the PI3K pathway is inhibited, some patients develop *KRAS* gene mutations (such as G12D and G13V) or *KRAS* protein overexpression, which compensates by activating the downstream MAPK pathway (ERK phosphorylation level increases 2-3 times) to maintain tumor cell activity 139. Clinical studies have shown that *KRAS* activation was detected in about 25%-30% of patients who failed apelelis treatment 140. After these patients received combination therapy with MEK inhibitors (such as trametinib), the ORR could recover to 15%-18% and the PFS could be prolonged to 4.0-4.5 months 141. In addition, *PTEN* loss can also lead to resistance to PI3K inhibitors: *PTEN* is a negative regulator of the PI3K/AKT pathway. Its gene deletion or protein expression downregulation leads to reduced PIP3 degradation. Even if PI3K is inhibited, AKT phosphorylation can still be maintained (p-AKT level increases by 1.8-2.2 times) 142. In patients treated with BKM120, the incidence of drug resistance in those with *PTEN* loss is 40%-45% higher than that in those with normal *PTEN*, and the time of drug resistance onset is 2-3 months earlier. For these patients, the combination of *PTEN* activators (such as disulfiram) can reduce AKT activity by 50%-55% and partially restore the sensitivity of tumor cells to PI3K inhibitors 143.

## Conclusion

This review systematically elucidates the regulatory mechanisms of gene mutations in the occurrence, development, and distant metastasis of cervical cancer. Through multidimensional analysis, it clarifies the action pathways of core mutated genes: genes such as *PDGFRA*, *TP53*, and *PIK3CA* collectively promote cervical cancer metastasis through mechanisms such as aberrant activation of signaling pathways (e.g., PI3K/Akt, MAPK), induction of epithelial-mesenchymal transition (EMT), enhanced tumor angiogenesis, and remodeling of the tumor microenvironment. The high metastasis association of low-frequency *PDGFRA* mutations, the dual regulation of cell cycle and apoptosis by *TP53* mutations, and the pathway activation effects of hotspot mutations in *PIK3CA* (e.g., exon9 E542K/E545K, exon20 H1047R) provide crucial clues for understanding the molecular mechanisms of metastasis.

In terms of clinical translational value, gene mutation detection overcomes the limitations of traditional imaging and tumor marker diagnosis. It can not only provide early warning of metastasis risk (e.g., the metastasis rate within 2 years for *PIK3CA*-mutated patients reaches 30%, significantly higher than the 10% for wild-type patients), but also guide personalized treatment. Tyrosine kinase inhibitors targeting *PDGFRA* (such as imatinib and sunitinib) and PI3K inhibitors targeting *PIK3CA* (such as apelips and BKM120) have shown preliminary efficacy, but resistance mechanisms (such as pathway compensatory activation and enhanced drug efflux pumps) and cost issues still need to be addressed. Furthermore, the synergistic effect of HPV integration and driver gene mutations (e.g., co-mutation of HPV16 E7 and *PIK3CA* accelerates metastasis), and the metastasis-regulating role of rare mutations such as *BRAF* (V600E) and *EGFR* (L858R) provide new directions for expanding therapeutic targets.

Future research should focus on the framework of "mutation-spatiotemporal-microenvironment-clinical intervention," and promote the application of "mutation-driven precision metastasis prevention" from theory to clinical practice by constructing AI metastasis risk prediction models through multi-center cohorts, developing ctDNA dynamic monitoring technology, and optimizing combination therapy regimens (such as PI3Kα inhibitors combined with PD-1 antibodies), ultimately improving the long-term survival rate and quality of life of patients with advanced cervical cancer.

## Discussion

This study provides theoretical and practical references for research on the association between cervical cancer gene mutations and distant metastasis. However, key analyses are needed from three aspects: mechanistic depth, technological limitations, and treatment challenges, to improve scientific rigor and clarify future directions.

### Core mutation gene mechanisms

From "Association" to "Causality" Current research confirms that *PDGFRA*, *TP53*, and *PIK3CA* mutations are associated with metastasis, but mechanistic gaps remain: the molecular interaction patterns of *PDGFRA* with low frequency (5%-10%) and high metastasis association (such as direct interaction with Rho GTPases and MMPs) and functional differences at different stages of metastasis are unclear, requiring verification of its "metastasis switch" role through CRISPR-edited PDX models combined with single-cell proteomics; the regulation of cell cycle and EMT by *TP53* mutations does not differentiate between exon5-8 hotspot mutations, and the interaction and co-metastasis mechanism with HPV E6/E7 proteins need further analysis, requiring verification using technologies such as dual-luciferase reporter genes; the activation intensity of *PIK3CA* hotspot mutations (exon9 E542K/E545K, exon20 H1047R) is not correlated with drug sensitivity, requiring the establishment of a "mutant subtype - pathway activity - drug IC50" model. Database-guided medication.

### Detection technology and clinical application: a breakthrough from "usable" to "reliable"

While gene mutation detection compensates for the shortcomings of traditional diagnosis, it has limitations: Conventional NGS has a detection rate of < 50% for low-abundance mutations in ctDNA (VAF < 1%). Ultrasensitive technologies such as ddPCR need to be introduced to establish a "VAF threshold - metastasis risk" relationship (e.g., verifying VAF≥0.5% as a *PDGFRA* warning threshold), and to construct a combined diagnostic panel of "gene mutation + epigenetic regulation" to increase diagnostic sensitivity from 85% to over 90%. Currently, the standards for "mutation abundance - clinical significance" are not uniform (e.g., differences in VAF interpretation for *TP53* mutations). Guidelines need to be developed in collaboration with international institutions based on tens of thousands of samples to clarify the risk stratification of mutation types and co-mutations.

### Treatment strategies

The Leap from "Effective" to "Long-Lasting" Targeted therapy faces resistance and cost issues: Resistance to *PDGFRA* and *PIK3CA* targeted drugs stems from secondary mutations (e.g., *PDGFRA* D842V), pathway compensation (e.g., MAPK activation), and microenvironmental influences (e.g., CAFs secreting IL-6). Dynamic ctDNA monitoring is needed to capture resistant clones, and specific inhibitors (e.g., MEK inhibitors combined with PI3K inhibitors) need to be developed. New combination therapies such as "targeted therapy + immunotherapy" (e.g., PI3K inhibitors reversing immunosuppression followed by PD-1 antibodies) are under-explored and require umbrella trials for validation. High treatment costs (e.g., apelelis at 15,000 RMB per month) limit widespread adoption, necessitating the development of low-cost multi-gene detection chips and the promotion of domestically produced targeted drugs for inclusion in medical insurance.

### Future research directions: multi-dimensional integrated planning

The research should revolve around the framework of "mutation - spatiotemporal - microenvironment - intervention": Basic research should construct double/triple mutant cell lines and conditional knockout mouse models to analyze the positive feedback of "mutant cells - microenvironment" (e.g., *PDGFRA* mutant cells recruit MDSCs); Clinical translation should establish multi-center cohorts (e.g., NCT-CERV-PROSPECT) and develop AI-based metastasis risk prediction models (incorporating mutation, pathology, and microenvironment indicators); Technological innovation should create a "drug resistance early warning - dynamic drug administration" platform to monitor drug resistance signals and adjust dosage through single-cell multi-omics to prolong progression-free survival of targeted therapy.

## Looking Forward

Over the next five years, cervical cancer metastasis research should be centered around a four-dimensional framework: "Mutation-Spatiotemporal-Microenvironment-Clinical Intervention":

Establish a multicenter prospective cohort (NCT-CERV-PROSPECT) covering three continents, integrating whole-exome, methylation, immune repertoire, and radiomics data to develop an AI-driven metastasis risk prediction model. The umbrella trial (UMBRELLA-CERVIX) was launched to evaluate the synergistic efficacy of a PI3Kα inhibitor combined with a PD-1 antibody in *PIK3CA*-mutant/immune-desert tumors, stratified by mutation subtype. Dynamic ctDNA response was prespecified as an early surrogate endpoint.

Using CRISPR biallelic gene editing and PDX-humanized mouse models, the researchers elucidated the mechanism by which *TP53*/*PIK3CA* co-mutations amplify PI3K signaling through the exosome-miR-21-*PTEN* axis, identifying druggable nodes.

Developing an international consensus standard for mutation abundance: Based on cross-validation using ddPCR and NGS, a VAF of 1% was established as a high-risk threshold, which will be incorporated into future FIGO staging updates.

Developing a "drug resistance early warning" platform: Leveraging single-cell multi-omics to monitor newly acquired mutations during treatment in real time, the researchers developed adaptive dosing algorithms to reduce the incidence of drug resistance and healthcare costs. Through the above-mentioned multi-dimensional closed-loop research, it is expected that by 2030, the leap from theory to clinic of "mutation-driven precise metastasis prevention" will be achieved, significantly improving the long-term survival and quality of life of patients with advanced cervical cancer.

## Figures and Tables

**Figure 2:**
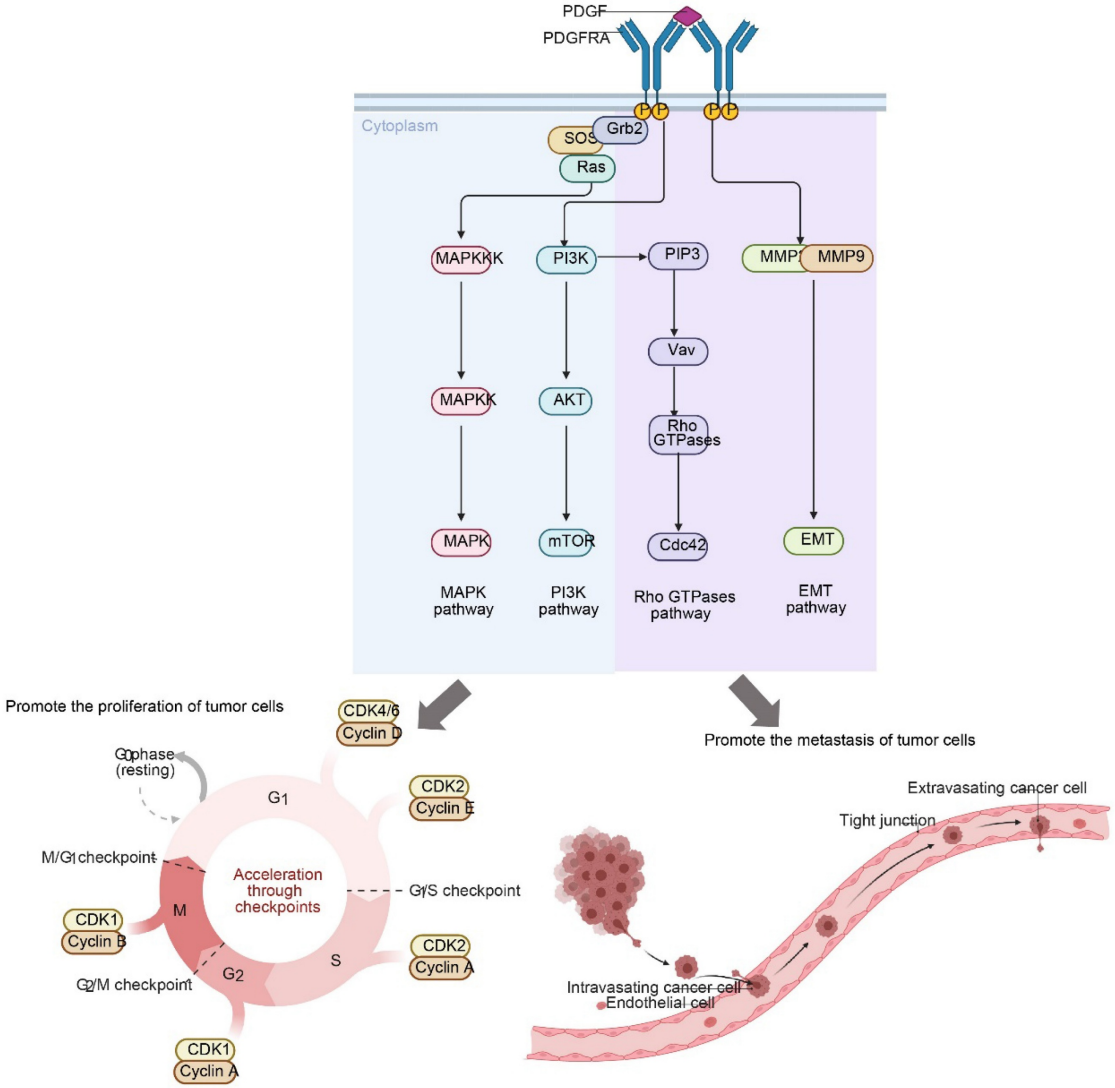
Mutations in PDGFRA promote the proliferation and metastasis of tumors. Upon ligand binding, PDGFRA activates key downstream signaling cascades, including the MAPK, PI3K-AKT-mTOR, Rho GTPases, and EMT pathways. These events collectively drive cell cycle acceleration (G1-S/M progression) and facilitate the metastatic process (intravasation and extravasation) by promoting cytoskeleton rearrangement and invasiveness.

**Figure 3:**
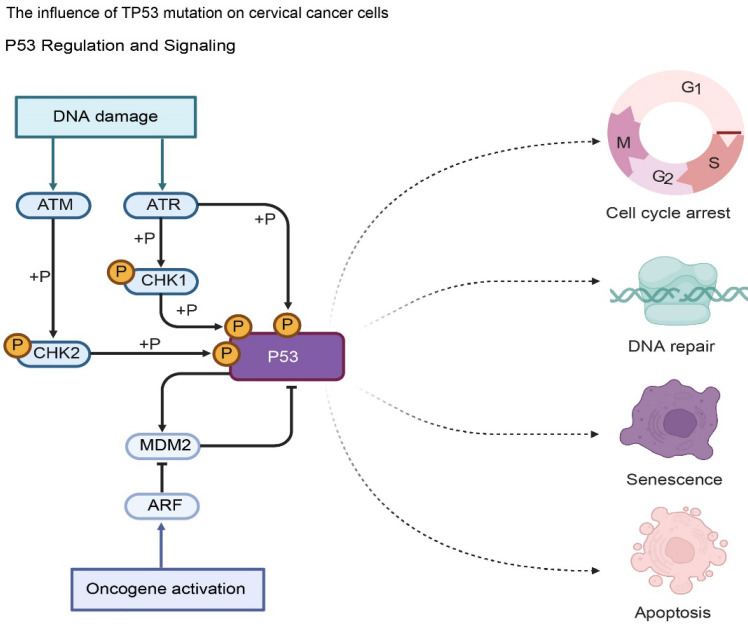
Impact of TP53 Mutations on Tumor Suppression Mechanisms. Under normal conditions, activated P53 triggers cell cycle arrest, DNA repair, or apoptosis in response to damage. TP53 mutations impair these tumor-suppressive functions, leading to the failure of checkpoints (ATM/ATR-CHK1/2 axis) and promoting the unchecked proliferation and malignant progression of cervical cancer cells.

**Figure 4:**
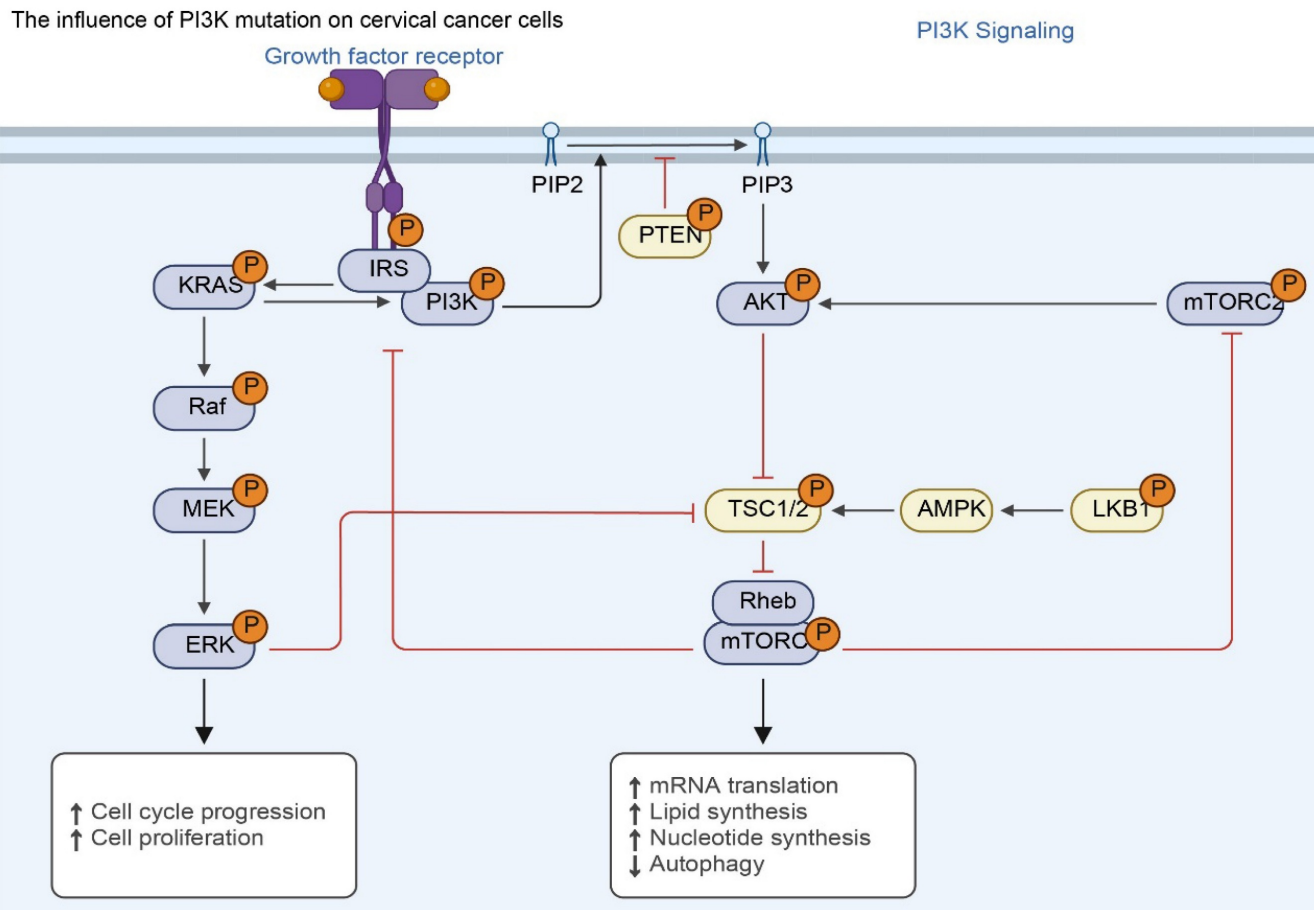
The effect of PI3K mutations on cervical cancer cells. Mutations in PIK3CA or loss of PTEN inhibition lead to constitutive activation of the PI3K-AKT-mTOR axis. This sustained signaling promotes malignant phenotypes—including uncontrolled cell cycle progression, protein/lipid synthesis, and inhibition of autophagy—independent of growth factor stimulation.

**Figure 5:**
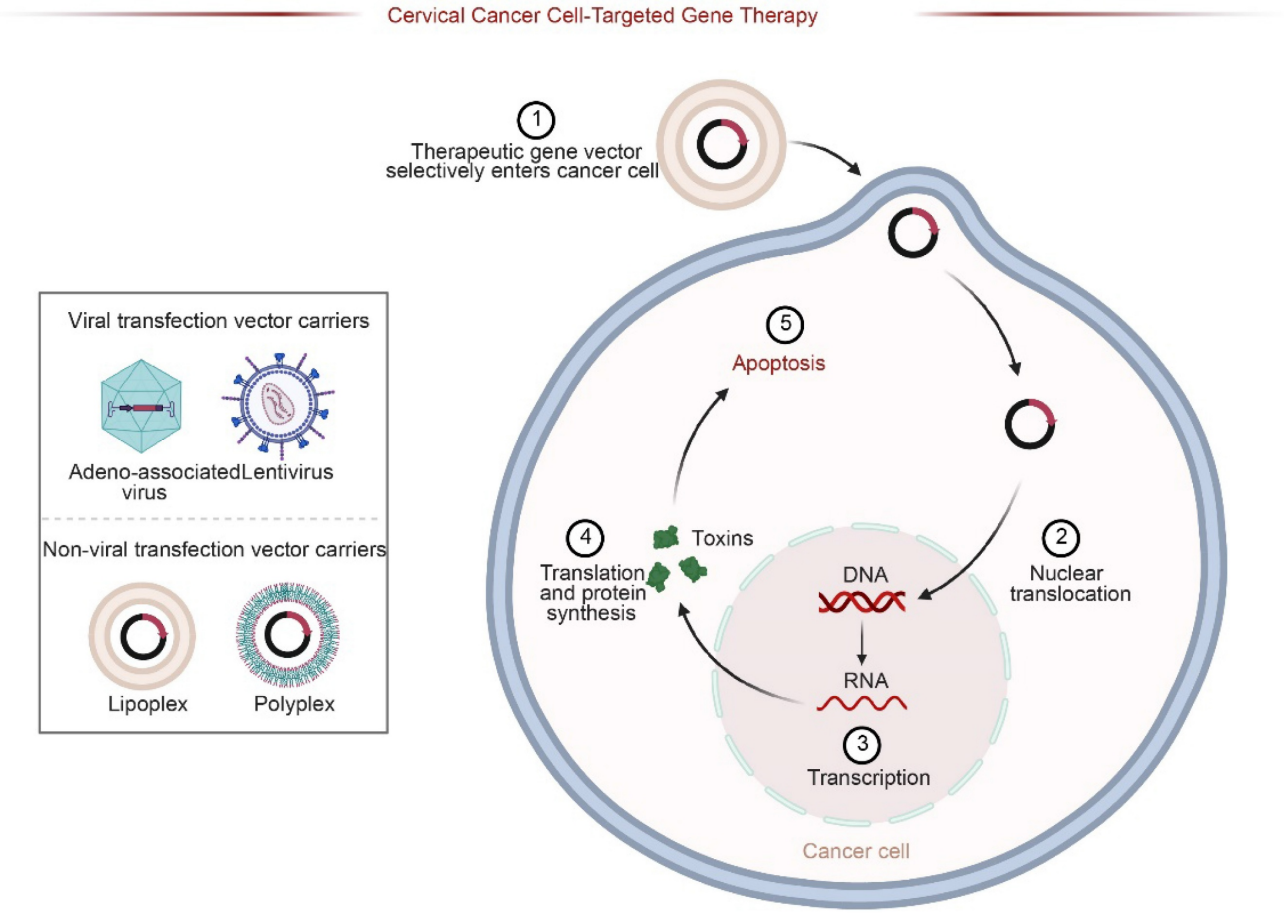
Schematic of Targeted Gene Therapy Strategies. Therapeutic genes are delivered via viral (e.g., Adeno-associated virus) or non-viral vectors (e.g., Lipoplexes). The vectors selectively enter cancer cells, undergo nuclear translocation, and express therapeutic proteins (e.g., toxins), ultimately inducing apoptosis in target cervical cancer cells.

**Figure 6:**
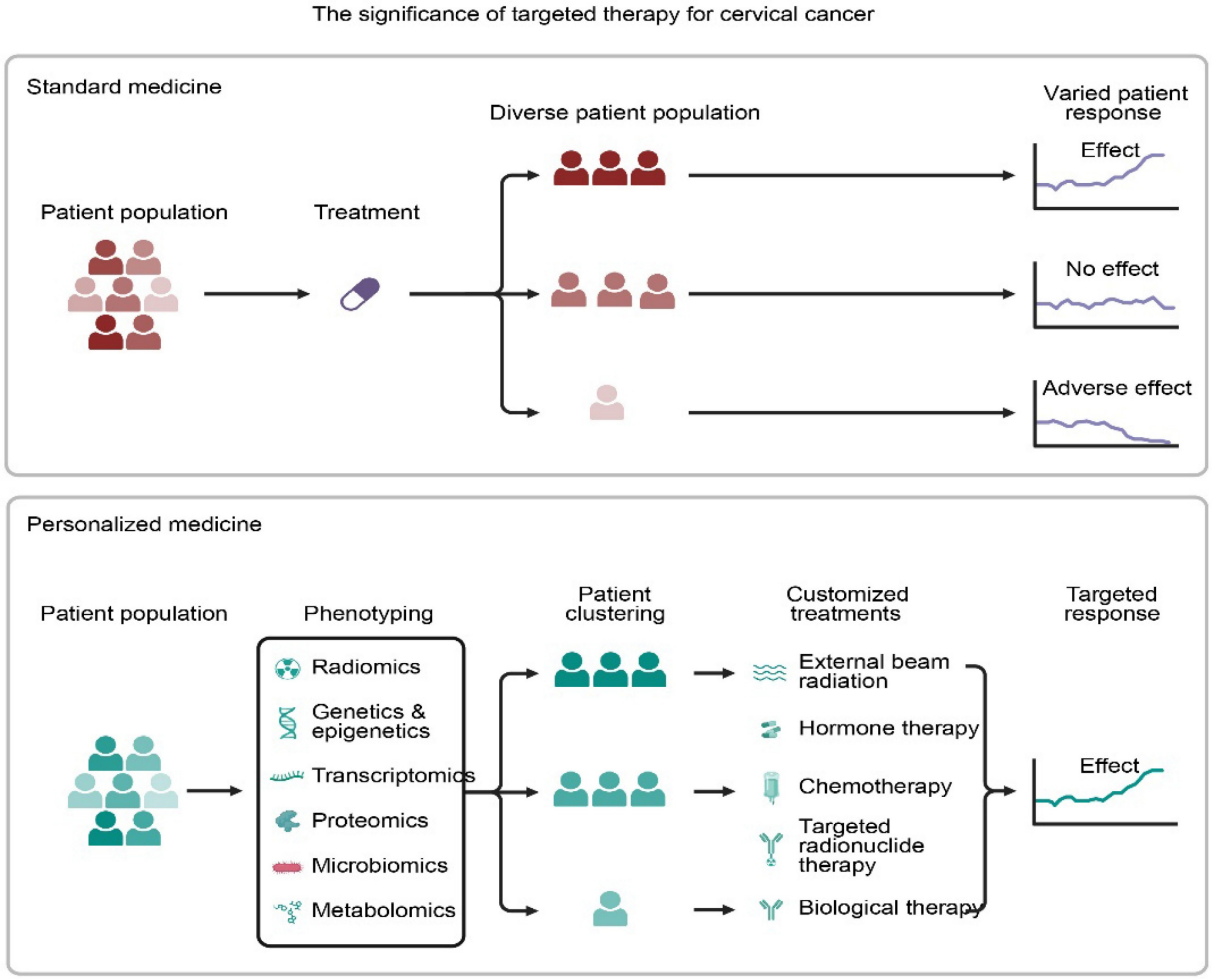
Paradigm Shift from Standard to Personalized Medicine. Standard treatment often results in variable patient outcomesdue to heterogeneity. In contrast, personalized medicine utilizes multi-omics phenotyping** (genetics, radiomics) to stratify patients, enabling customized therapeutic strategies that enhance treatment consistency and efficacy.

**Table 1:** Summary of key molecular events and clinical implications in cervical cancer metastasis

Primary Category	Secondary Category	Core Content (Refined)
Metastatic Pathways	Direct Spread (70%-80%)	• Most common mode; invades vaginal wall, uterine cavity, and pelvic wall • Symptoms: Irregular bleeding, lumbosacral pain • Risk factors: Tumor size, location, and differentiation
	Lymphatic Metastasis (20%-30%)	• Progression: Level I (pelvic) → Level II (para-aortic) nodes • Risk factors: Advanced stage, adenocarcinoma subtype, poor differentiation
	Hematogenous Metastasis (5%-10%)	• Sites: Lungs, liver, bones, brain • Prognosis: Rare but associated with poor outcomes
Gene Mutations	PDGFRA	• Role: Activates MAPK/PI3K pathways; upregulates Rho GTPases/MMPs • Impact: Linked to lymph node metastasis; increases cisplatin resistance
	TP53	• Mechanism: Impairs cell cycle checkpoints (p21) and apoptosis (Bax/Bcl-2) • Effect: Promotes EMT and invasion; associated with 30-50% of cases
	PIK3CA	• Hotspots: Exon 9 & 20; constitutively activates Akt/mTOR • Clinical: Higher mutation rate in metastatic patients (30%) vs. non-metastatic (10%)
	Other (*BRAF, EGFR, KRAS, PTEN*)	• *BRAF/KRAS*: Activate MAPK pathway; *BRAF* V600E linked to lung metastasis • *PTEN*: Loss leads to uncontrolled PI3K signaling and MMP upregulation
Clinical Application	Early Identification	• Prognostic Value: *PIK3CA* mutations predict a significantly higher 2-year metastasis rate (30% vs 10%)
	Diagnosis & Treatment	• Accuracy: Gene detection combined with imaging improves specificity (e.g., distinguishing lung metastasis) • Targeted Therapy: Supports use of TKIs (Imatinib) or PI3K inhibitors (Alpelisib) for precise intervention
